# Relationships of serum high-sensitivity C-reactive protein and body size with insulin resistance in a Japanese cohort

**DOI:** 10.1371/journal.pone.0178672

**Published:** 2017-06-02

**Authors:** Hirokazu Uemura, Sakurako Katsuura-Kamano, Miwa Yamaguchi, Tirani Bahari, Masashi Ishizu, Miho Fujioka, Kokichi Arisawa

**Affiliations:** Department of Preventive Medicine, Institute of Biomedical Sciences, Tokushima University Graduate School, Japan; German Diabetes Center, Leibniz Center for Diabetes Research at Heinrich Heine University Düsseldorf, GERMANY

## Abstract

**Background:**

Impacts of chronic systemic inflammation and body size and their interaction effect on insulin resistance in Asian populations, in whom obesity is less common, are not fully understood. This study evaluated combined relationships of systemic inflammation and body size with insulin resistance in a Japanese cohort.

**Methods:**

We analyzed cross-sectional data from 1,074 eligible subjects (536 men and 538 women) aged 35–69 years who participated in the baseline survey of a cohort study in Tokushima Prefecture, Japan. Systemic inflammation level was assessed by serum high-sensitivity C-reactive protein (hs-CRP), and the degree of insulin resistance and beta-cell function were evaluated by the homeostasis model assessment insulin resistance (HOMA-IR) and beta-cell function (HOMA-β), respectively. Overweight and obesity were defined as a body mass index (BMI) of 23.0–24.9 kg/m^2^ and ≥25.0 kg/m^2^, respectively. Associations between serum hs-CRP (assessed as quartiles and additionally continuous values after log-transformation) and indices of glucose homeostasis were analysed adjusting for probable covariates, including BMI (quartiles). Combined associations of serum hs-CRP (≤median, >median) and body size (normal, overweight, obese) with insulin resistance as well as their interaction effect on insulin resistance were also evaluated.

**Results:**

Serum hs-CRP was dose-dependently associated with HOMA-IR, but not HOMA-β, after adjustment for probable covariates, including BMI. Subjects with obesity and elevated serum hs-CRP (>median) showed a high multivariable-adjusted HOMA-IR value of 1.32 (95% confidence interval (CI) 1.23, 1.41) compared with subjects with normal BMI and low serum hs-CRP (≤median) whose multivariable-adjusted HOMA-IR value was 1.14 (95% CI 1.06, 1.21). The interaction effect between body size (normal, overweight, obese) and serum hs-CRP (≤median, >median) on HOMA-IR was significant (*P* for interaction <0.001).

**Conclusions:**

Our study suggests that elevated systemic inflammation is dose-dependently associated with increased insulin resistance, independent of the known risk factors, in a Japanese population. Concomitant obesity and elevated systemic inflammation may synergistically contribute to increased insulin resistance.

## Introduction

The prevalence of type 2 diabetes has been increasing worldwide [1]. Many patients with type 2 diabetes suffer from microvascular and macrovascular complications, such as retinopathy, nephropathy, neuropathy, heart disease, and stroke [2]. Insulin resistance is a condition in which the peripheral tissues of the human body becomes resistant to the action of insulin. Insulin resistance is strongly associated with the development of type 2 diabetes. Therefore, it is of interest to identify the various risk factors for developing insulin resistance, so that preventive measures can be developed.

Obesity is well-recognized as a key risk factor for various chronic diseases, including insulin resistance and type 2 diabetes [3,4]. Although obesity is less common in Japan than in Western countries, the prevalence of type 2 diabetes in Japan is rather high; the estimated prevalence rate in Japanese adults by International Diabetes Federation (IDF) was 7.6% in 2013 [5]. Low-grade systemic inflammation has received much attention as a key player in the pathogenesis of various diseases, such as cardiovascular disease [6,7] and type 2 diabetes [8,9]. C-reactive protein (CRP) is produced by the liver in response to inflammation in the body [10]. Blood levels of high-sensitivity CRP (hs-CRP) have been used as a biomarker of low-grade systemic inflammation. Many studies have demonstrated independent relationships between various inflammatory markers, such as hs-CRP and interleukin (IL)-6, and the development of type 2 diabetes [8,9,11]. However, a meta-analysis suggested that hs-CRP may not be an independent risk factor for developing type 2 diabetes [12]. There are also reports demonstrating an association between circulating hs-CRP and insulin resistance [13,14]. However, few studies have evaluated the combined associations of body size and low-grade systemic inflammation with insulin resistance in Asian populations, which are known to have lower rates of obesity.

We have conducted a prospective cohort study from 2008 in Tokushima Prefecture, Japan. In the present study, using the baseline data (cross-sectional data) from this Japanese cohort, we evaluated the relationships of body size and low-grade systemic inflammation with insulin resistance.

## Materials and methods

### Study subjects

The present study included 1,266 participants, aged 35–69 years, who were enrolled in the baseline survey of a prospective cohort study from January 2008 to February 2013 in Tokushima Prefecture, Japan, which is performed as part of the Japan Multi-Institutional Collaborative Cohort (J-MICC) Study. Details of the J-MICC Study have been reported elsewhere [15]. Briefly, the J-MICC Study aims to examine the associations of lifestyle and genetic factors, as well as their interactions with lifestyle-related diseases. Subjects in the present study were recruited in two ways. The first group consisted of 570 participants who received health examinations at the Tokushima Prefectural General Health Check-up Center from January 2008 to November 2011. The second group consisted of 696 general inhabitants of Tokushima city and neighboring towns who were recruited through a distributed leaflet and attended health check-ups, which were performed by our research team from July 2012 to February 2013.

Of the total of 1,266 participants (637 men and 629 women), 192 participants (101 men and 91 women) were excluded if they met any of the following criteria (with possible overlap): subjects with self-reported previous history of ischemic heart disease (n = 29), stroke (n = 14), or medical treatment for diabetes (n = 50); subjects with missing data for fasting plasma glucose (FPG) and/or insulin (n = 9), diabetes treatment (n = 0), or any clinical parameters included in the multivariable-adjusted models (n = 31); subjects whose estimated daily energy intake was extremely high (>4000 kcal/day) or low (<1000 kcal/day) (n = 11); and subjects whose serum hs-CRP values were missing (n = 5) or ≥10 mg/L (since acute inflammatory status could not be ruled out) (n = 15); subjects with a history of rheumatoid arthritis accompanied by systemic inflammation (n = 4); or, those with regular use of anti-inflammatory analgesics that might affect hs-CRP values (n = 48). Data from the eligible 1,074 subjects (536 men and 538 women) were used for cross-sectional analyses.

All participants in the J-MICC Study provided written informed consent prior to participation. This study was conducted according to the principle set forth by the Declaration of Helsinki. The Ethics Committees of Nagoya University School of Medicine (the affiliation of the former principal investigator, Nobuyuki Hamajima), Aichi Cancer Center (the affiliation of the current principal investigator, Hideo Tanaka), and Tokushima University Graduate School approved the protocol of this study.

### Questionnaire

Information on individual lifestyle characteristics, including medical history, medications, smoking and drinking habits, leisure-time exercise, and dietary habits were obtained through a structured self-administered questionnaire. All the responses were checked by trained staff at the time of the survey [16,17].

Leisure-time exercise was divided into three categories, light, moderate, and heavy, based on the intensity of the exercise: 3.4, 7.0, and 10.0 metabolic equivalents (METs), respectively. The level of each exercise category was calculated by multiplying the frequency of each exercise activity by its duration. These values were then summed for all activities to estimate the degree of leisure-time exercise. Values are expressed as MET-hours/week, as described previously [16,17].

Diet was evaluated using a validated short food frequency questionnaire (FFQ) [18–21]. The FFQ inquired about the intake of 47 food and beverage items over the past year. Daily energy intake was estimated using a program developed by the Department of Public Health, Nagoya City University School of Medicine [18,19].

### Measurements and assessment of overweight, obesity and glucose homeostasis

Body height, body weight, waist circumference, and FPG were measured during the health check-ups at baseline. BMI was calculated as weight (kg) divided by the square of height (m). Venous blood was drawn from each participant, and serum was separated within 3 hours and stored at– 80°C. Fasting insulin and hs-CRP were measured from stored serum samples at an external laboratory (BML Inc., Tokyo, Japan).

Because BMIs in Asian populations are generally smaller than those in Western populations [22,23], the World Health Organization (WHO) [24], the International Association for the Study of Obesity [25,26], and the International Obesity Task Force [25,26] have proposed that the BMI cutoff levels for overweight and obesity for Asians should be lower than the international classification criteria. In Japan, a BMI ≥25 kg/m^2^ is the proposed cutoff level for obesity [27–29]. According to these proposals, overweight for Japanese subjects in this study was defined as BMI 23.0–24.9 kg/m^2^ and obesity was defined as BMI ≥25.0 kg/m^2^.

The degree of insulin resistance and beta-cell function were evaluated by the homeostasis model assessment insulin resistance (HOMA-IR) and beta-cell function (HOMA-β), respectively [30]. These indices were calculated according to the following formulas:

HOMA-IR=fastinginsulin(μU/mL)×FPG(mg/dL)/405.

HOMA-β=fastinginsulin(μU/mL)×360/[FPG(mg/dL)-63].

### Statistical analyses

Continuous variables are expressed as mean ± SD or median (25^th^ percentile, 75^th^ percentile). Categorical variables are expressed as the number (%). ANOVA, Kruskal–Wallis tests, or chi-square tests were used to evaluate differences in the baseline characteristics of subjects according to serum hs-CRP levels (quartiles). We first evaluated the associations between serum hs-CRP (quartiles) and indices of glucose homeostasis (fasting insulin, FPG, HOMA-IR, and HOMA-β) by general linear models while adjusting for the following covariates: 1) age (continuous) and sex (model 1); 2) age, sex, recruit group (binary), smoking status (current, past, never), current alcohol drinker (no, yes), leisure-time exercise (MET-hours/week; quartiles), and daily energy intake (kcal/day; quartiles) (model 2); 3) the covariates in model 2 plus BMI (kg/m^2^; quartiles) (model 3); and 4) the covariates in model 2 plus waist circumference (cm; quartiles) (model 4). The dose-dependent relationships between serum hs-CRP and insulin resistance were assessed by assigning the median hs-CRP value of each hs-CRP quartile to the respective quartile in the general linear models. We additionally assessed the associations between serum hs-CRP levels and indices of glucose metabolism by linear regression using serum hs-CRP as a continuous variable after log-transformation. Next, we evaluated the combined associations of serum hs-CRP (≤median, >median) and body size (normal, overweight, obese) with HOMA-IR by similar general linear models. The interaction effects between serum hs-CRP (≤median, >median) and body size (normal, overweight, obese) on insulin resistance were evaluated by including interaction terms in the models. Since the values of FPG, fasting insulin, HOMA-IR, and HOMA-β followed right-skewed distributions, they were log-transformed before inclusion in the general linear models and linear regression models.

All calculations and statistical tests were performed using SAS version 9.4 (SAS Institute Inc., Cary, NC, USA). Statistical tests were based on 2-sided probabilities, and the level of significance was set at *P* <0.05.

## Results

Table 1 shows the baseline characteristics of subjects according to serum hs-CRP quartiles. The percentage of men and current smokers, BMI, waist circumference, and the prevalence of obesity and overweight were higher with higher serum hs-CRP levels. Similarly, FPG, fasting insulin, and HOMA-IR values dose-dependently increased as serum hs-CRP levels increased.

**Table 1:** Clinical characteristics of the subjects according to serum hs-CRP quartiles.

	Serum hs-CRP				
	Q1 (≤0.15mg/L)	Q2 (>0.15∼0.30mg/L)	Q3 (>0.30∼0.64mg/L)	Q4 (>0.64mg/L)	*P*
Number ^c^	271 (25.2)	268 (25.0)	267 (24.9)	268 (25.0)	
Men ^c^	94 (17.5)	132 (24.6)	149 (27.8)	161 (30.0)	<0.001
Recruit group ^c^					
Health Check-up Center	107 (39.5)	128 (47.8)	127 (47.6)	132 (49.3)	0.095
Participants by leaflet	164 (60.5)	140 (52.2)	140 (52.4)	136 (50.7)	
Age (years) ^a^	50.0 ± 9.8	52.5 ± 9.3	53.9 ± 9.6	53.1 ± 10.1	<0.001
BMI (kg/m^2^) ^b^	21.4 (19.6, 23.0)	22.4 (20.6, 24.3)	23.4 (21.7, 25.5)	24.8 (22.5, 27.3)	<0.001
Waist circumference (cm) ^b^	76.0 (70.0, 82.0)	80.5 (74.5, 86.0)	83.0 (76.0, 89.0)	87.0 (80.3, 93.0)	<0.001
Smoking habit ^c^					
Current	35 (12.9)	35 (13.1)	46 (17.2)	60 (22.4)	0.014
Past	64 (23.6)	66 (24.6)	72 (27.0)	73 (27.2)	
Never	172 (63.5)	167 (62.3)	149 (55.8)	135 (50.4)	
Alcohol drinking ^c^					
Current	135 (49.8)	153 (57.1)	143 (53.6)	155 (57.8)	0.220
Never or Past	136 (50.2)	115 (42.9)	124 (46.4)	113 (42.2)	
Leisure-time exercise (MET-hours/week) ^b^	5.10 (0.43, 15.75)	7.65 (1.28, 21.25)	7.65 (1.28, 23.45)	6.25 (1.28, 17.93)	0.088
Energy intake (kcal/day) ^b^	1584 (1418, 1827)	1702 (1520, 1896)	1651 (1461, 1872)	1713 (1517, 1957)	<0.001
Serum hs-CRP (mg/L) ^b^	0.10 (0.08, 0.13)	0.22 (0.19, 0.26)	0.43 (0.36, 0.51)	1.15 (0.84, 1.85)	<0.001
Fasting plasma glucose (mg/dL) ^b^	90 (84, 96)	91 (86, 98)	92 (87, 99)	95.5 (89, 103)	<0.001
Fasting insulin (μU/mL) ^b^	4.2 (3.1, 5.7)	4.7 (3.4, 6.4)	5.0 (3.6, 7.6)	6.5 (4.1, 10.4)	<0.001
HOMA-IR ^b^	0.91 (0.68, 1.28)	1.09 (0.75, 1.50)	1.16 (0.80, 1.80)	1.52 (0.96, 2.55)	<0.001
HOMA-β ^b^	60 (41, 81)	59 (44, 80)	62 (45, 91)	70 (45, 105)	0.002
Prevalence ^c^					
Obesity (BMI ≥25.0 kg/m^2^)	28 (10.3)	52 (19.4)	81 (30.3)	130 (48.5)	<0.001
Overweight (BMI 23.0–24.9 kg/m^2^)	67 (24.7)	113 (42.2)	156 (58.4)	185 (69.0)	<0.001

^a^ Mean ± SD.

^b^ Median (25%, 75%).

^c^ Number (%)

hs-CRP, high-sensitivity C-reactive protein; BMI, body mass index; MET, metabolic equivalent

HOMA-IR, homeostasis model assessment insulin resistance; HOMA-β, homeostasis model assessment beta-cell function

Differences are analyzed by ANOVA^a^, Kruskal–Wallis test^b^, or chi-square test^c^.

### Associations between serum hs-CRP and indices of glucose homeostasis

As presented in Table 2, elevated serum hs-CRP was dose-dependently associated with increased fasting insulin, FPG, and HOMA-IR, after adjustment for the multivariable covariates in model 2 (all *P* for trend <0.001). After additional adjustment for BMI (model 3) or waist circumference (model 4), associations were slightly attenuated, but remained significant. In contrast, elevated serum hs-CRP was dose-dependently associated with increased HOMA-β in model 2; however, this association was attenuated and became non-linear after additional adjustment for BMI (model 3) or waist circumference (model 4).

**Table 2:** Associations between serum hs-CRP levels and indices of glucose metabolism.

	Serum log(hs-CRP) (continuous)			Serum hs-CRP (mg/L, quartiles)				
				Q1 (≤0.15)	Q2 (>0.15∼0.30)	Q3 (>0.30∼0.64)	Q4 (>0.64)	*P for trend*
	β estimate^a^	SE	*P value*	Mean (95% CI)^b^	Mean (95% CI)^b^	Mean (95% CI)^b^	Mean (95% CI)^b^	
Fasting insulin (μU/mL)								
Model 1	0.130	0.016	<0.001	4.32 (4.04, 4.62)	4.74 (4.44, 5.06)	5.30 (4.96, 5.66)	6.32 (5.92, 6.75)	<0.001
Model 2	0.132	0.016	<0.001	4.36 (4.07, 4.67)	4.78 (4.46, 5.13)	5.36 (5.01, 5.74)	6.39 (5.97, 6.84)	<0.001
Model 3	0.038	0.015	0.011	4.95 (4.65, 5.27)	5.07 (4.76, 5.39)	5.20 (4.89, 5.53)	5.56 (5.22, 5.91)	0.007
Model 4	0.055	0.015	<0.001	4.93 (4.62, 5.26)	5.07 (4.76, 5.41)	5.34 (5.02, 5.68)	5.79 (5.44, 6.17)	<0.001
Fasting plasma glucose (mg/dL)								
Model 1	0.020	0.003	<0.001	91.8 (90.6, 93.1)	92.5 (91.3, 93.8)	93.2 (92.0, 94.5)	97.0 (95.7, 98.3)	<0.001
Model 2	0.021	0.003	<0.001	92.0 (90.7, 93.3)	92.4 (91.2, 93.7)	93.4 (92.1, 94.7)	97.3 (95.9, 98.6)	<0.001
Model 3	0.014	0.003	<0.001	92.9 (91.6, 94.2)	92.9 (91.6, 94.2)	93.3 (92.0, 94.5)	96.1 (94.8, 97.4)	<0.001
Model 4	0.015	0.003	<0.001	92.9 (91.7, 94.2)	93.0 (91.7, 94.2)	93.4 (92.1, 94.7)	96.4 (95.1, 97.7)	<0.001
HOMA-IR								
Model 1	0.151	0.018	<0.001	0.98 (0.91, 1.05)	1.08 (1.01, 1.16)	1.22 (1.14, 1.31)	1.51 (1.41, 1.63)	<0.001
Model 2	0.153	0.017	<0.001	0.99 (0.92, 1.07)	1.09 (1.01, 1.18)	1.24 (1.15, 1.33)	1.53 (1.43, 1.65)	<0.001
Model 3	0.053	0.016	0.001	1.14 (1.06, 1.21)	1.16 (1.09, 1.24)	1.20 (1.12, 1.28)	1.32 (1.23, 1.41)	<0.001
Model 4	0.069	0.017	0.001	1.13 (1.06, 1.21)	1.16 (1.09, 1.25)	1.23 (1.15, 1.32)	1.38 (1.29, 1.47)	<0.001
HOMA-β								
Model 1	0.071	0.016	<0.001	56.1 (52.6, 59.8)	60.1 56.4, 64.0)	65.8 (61.7, 70.0)	70.0 (65.7, 74.6)	<0.001
Model 2	0.069	0.016	<0.001	56.4 (52.8, 60.3)	61.0 (57.0, 65.2)	66.3 (62.0, 70.8)	70.3 (65.9, 75.1)	<0.001
Model 3	-0.001	0.015	0.943	62.1 (58.3, 66.2)	63.6 (59.8, 67.8)	64.6 (60.7, 68.8)	63.5 (59.6, 67.6)	0.837
Model 4	0.011	0.016	0.480	61.9 (58.0, 66.0)	63.7 (59.7, 67.9)	66.0 (62.0, 70.3)	65.4 (61.4, 69.7)	0.329

hs-CRP, high-sensitivity C-reactive protein; Q1, first quartile; Q2, second quartile; Q3, third quartile; Q4, fourth quartile; SE, standard error; CI, confidence interval

HOMA-IR, homeostasis model assessment insulin resistance; HOMA-β, homeostasis model assessment beta-cell function

Model 1: adjusted for age and sex

Model 2: adjusted for age, sex, recruit group, smoking status, current alcohol drinking, leisure-time exercise, and daily energy intake

Model 3: adjusted for the covariates in model 2 plus body mass index

Model 4: adjusted for the covariates in model 2 plus waist circumference

^a^ β estimates of the indexes of glucose metabolism after these indexes were log-transformed.

^b^ Geometric adjusted means (95% CI) of the indexes of glucose metabolism.

### Combined associations of body size and serum hs-CRP levels with HOMA-IR

As shown in Table 3, the adjusted mean of the HOMA-IR did not differ between the high (≥median) and low (<median) hs-CRP categories in normal weight subjects. In contrast, the adjusted mean of the HOMA-IR was about 1.5 times higher in the high hs-CRP category compared to the low hs-CRP category in obese subjects. The interaction effect between body size (normal, overweight, obese) and serum hs-CRP (≤median, >median) on HOMA-IR was significant (*P* for interaction <0.001, model 2).

**Table 3:** Combined associations of body size and serum hs-CRP levels with HOMA-IR.

		Body size			
		Normal	Overweight	Obesity	* *P*_interaction_
		(BMI<23.0 kg/m^2^)	(BMI 23.0~24.9 kg/m^2^)	(BMI>25.0 kg/m^2^)	
		Geometric adjusted means (95% confidence intervals) of HOMA-IR			
Model 1					
	Hs-CRP				
	≤median	0.92 (0.87, 0.97)	1.16 (1.04, 1.28)	1.42 (1.27, 1.60)	<0.001
	>median	0.91 (0.84, 0.98)	1.27 (1.16, 1.39)	2.10 (1.95, 2.25)	
Model 2					
	Hs-CRP				
	≤median	0.93 (0.87, 0.98)	1.16 (1.05, 1.29)	1.44 (1.28, 1.63)	<0.001
	>median	0.93 (0.86, 1.00)	1.28 (1.17, 1.41)	2.13 (1.98, 2.29)	

hs-CRP, high-sensitivity C-reactive protein; HOMA-IR,homeostasis model assessment-insulin resistance

The median of hs-CRP was 0.30 mg/L.

Model 1: adjusted for age and sex

Model 2: adjusted for age, sex, recruit group, smoking habit, current alcohol drinking, leisure-time exercise, and daily energy intake

* P values for interaction between body size (3 categories; normal, overweight, and obesity) and serum hs-CRP (dichotomous; ≤median, >median) on HOMA-IR

## Discussion

The current study showed that elevated serum hs-CRP, a biomarker of systemic inflammation, was dose-dependently associated with an increased degree of insulin resistance, after adjustment for traditional risk factors in a Japanese population. There was a significant interaction effect between body size and serum hs-CRP on insulin resistance. Moreover, the association of serum hs-CRP with insulin resistance was stronger in obese subjects compared to normal weight subjects.

The prevalence of type 2 diabetes has increased globally, and is considered a major worldwide health problem [1]. Insulin resistance is a high-risk condition for the development of type 2 diabetes and its prevalence is thought to be increasing in Japan. Although obesity is an established risk factor for insulin resistance, obesity is less common in Japan than in Western countries. In general, Asian populations have smaller BMIs than Western populations [22,23]. As such, the BMI cutoff levels for overweight and obesity for Asians are lower than international classification criteria. For example, a BMI ≥25 kg/m^2^ is the proposed cutoff level for obesity in Japanese individuals, rather than the international classification criteria of a BMI ≥30 kg/m^2^ [27–29]. It is postulated that Asians have a limited innate capacity of insulin secretion [31] and that basal insulin secretion is lower in Japanese individuals than in Westerners. Therefore, Japanese people may be more susceptible to insulin resistance with small disruptions in glucose homeostasis that occur, for example, after modest weight gain.

Chronic systemic inflammation has received much attention as a key player in the pathogenesis of various diseases, such as cardiovascular disease [6,7], type 2 diabetes [8,9], and insulin resistance [13,14]. We observed that elevated serum hs-CRP was dose-dependently associated with an increased degree of insulin resistance, assessed by HOMA-IR, after adjustment for probable covariates (model 2). This association was attenuated after additional adjustment for BMI (model 3), though it remained significant. When waist circumference, which estimates visceral fat volume, was adjusted instead of BMI (model 4), the results did not substantially alter. Systemic inflammation is suggested to be elevated in obese subjects. The age-and-gender-adjusted partial correlation coefficients of serum hs-CRP with BMI and waist circumference in our subjects were significant but moderate (r = 0.262, *P* <0.001 and r = 0.245, *P* <0.001, respectively). Therefore, the relationship between elevated serum hs-CRP and increased insulin resistance may be partially explained by increased BMI and/or visceral adiposity; however, other mechanisms that lead to a rise in serum hs-CRP can contribute to insulin resistance. Although the mechanisms underlying the relationship between elevated serum hs-CRP and insulin resistance have not been fully elucidated, some plausible mechanisms have been proposed. An animal study using rats provided *in vivo* evidence that human CRP plays an active role in inducing hepatic insulin resistance, which is at least partially achieved through impairment in the insulin signaling pathway [32]. Therefore, CRP may adversely affect insulin sensitivity through direct action on the liver. Additionally, tumor necrosis factor alpha (TNF-α) and IL-6, which are pro-inflammatory cytokines secreted by adipose tissue, can stimulate CRP production in the liver [33]. In particular, TNF-α induces insulin resistance [34]. An *in vitro* study in mice found that chronic exposure to IL-6 inhibits insulin receptor signal transduction in primary hepatocytes [35]. Therefore, increased TNF-α and/or IL-6 secretion could explain the relationship between elevated serum hs-CRP and insulin resistance.

In contrast to the findings for HOMA-IR, dose-dependent positive associations between serum hs-CRP levels and HOMA-β were attenuated after additional adjustment for BMI or waist circumference. These findings were almost concordant with previous reports [36,37]. Festa et al. [36] reported that inflammation in the prediabetic state was related to increased insulin resistance rather than decreased insulin secretion. Herder et al. [37] have recently reported that higher hs-CRP and interleukin (IL)-6 were associated with increases in fasting insulin and insulin resistance and IL-6 (but not hs-CRP) was associated with HOMA-β. Although we have no data on IL-6, it has been reported that IL-6 can stimulate insulin secretion through glucagon-like peptide-1 (GLP-1) production and secretion in animal models of type 2 diabetes [38]. Because persons with medical treatment for diabetes were excluded in the current study, most of our subjects could be regarded as non-diabetic and their glucose homeostasis was maintained. In such subjects, dose-dependent association between elevated serum hs-CRP and increased HOMA-β after adjusting for probable covariates (model 2) might reflect a compensatory response of islet beta-cells against decreased insulin action. Considering that associations between serum hs-CRP levels and HOMA-β were attenuated after adjustment for BMI or waist circumference, body size may be strongly associated with islet beta-cell function, and systemic inflammation may not be independently associated with islet beta-cell function in this Japanese population.

We found that the combination of obesity and elevated systemic inflammation was associated with a greater degree of insulin resistance, and that the interaction effect between body size and serum hs-CRP on insulin resistance was significant. Obesity and elevated serum hs-CRP synergistically increase insulin resistance, and obesity may affect insulin resistance through both inflammation dependent and independent pathways. Further studies are required to clarify these mechanisms and establish causal effects of systemic inflammation and obesity on insulin sensitivity and resistance, especially in humans.

Our study has several limitations. First, because of the cross-sectional study design, a causal relationship between serum hs-CRP and insulin resistance cannot be established. Second, information about medical history and other lifestyle factors were self-reported; therefore, non-differential misclassification may have occurred. Third, although information was collected on several potential confounding factors and was adjusted for in analyses, residual confounding may exist. Fourth, although HOMA models are widely used to assess insulin resistance and beta-cell function because they require only fasting glucose and insulin levels, HOMA-IR is less accurate for assessing insulin resistance than euglycemic hyperinsulinemic clamp method. HOMA-β estimates fasting beta-cell function and cannot assess dynamic beta-cell function. Moreover, HOMA models assume the linear insulin response to increasing glucose levels and the use of these models should be carefully considered in subjects with fasting hyperglycemia. However, persons with medical treatment for diabetes were excluded in this study and the number of subjects whose fasting plasma glucose ≥140 mg/dL was only 8 (0.74%). Finally, because all subjects were Japanese, our results may not be applicable to other ethnic populations.

In conclusion, our study demonstrates that elevated systemic inflammation, measured by serum hs-CRP, was dose-dependently associated with increased insulin resistance after adjustment for traditional risk factors, including BMI, in a Japanese population. Concomitant obesity and systemic inflammation might synergistically contribute to insulin resistance. Large prospective studies are required to confirm the observed associations and to establish causality between systemic inflammation, obesity, and insulin resistance.
